# Supplementary material for: Calculation of Permeability Coefficients from Solute Equilibration Dynamics: An Assessment of Various Methods
Source: Membranes (Basel). 2022 Feb 23;12(3):254. doi: 10.3390/membranes12030254 (PMC8951150; doi:10.3390/membranes12030254)
Supplement: Supplementary file 1 [file membranes-12-00254-s001.zip › membranes-1593294-supplementary.pdf]

membranes

MDPI

Supplementary Materials for

Calculation of Permeability Coefficients from Solute Equilibration Dynamics: An Assessment of Various Methods

Margarida M. Cordeiro [1,2], Armindo Salvador [1,3,4,*] and Maria João Moreno [1,2,*]

[1] Coimbra Chemistry Center-Institute of Molecular Sciences (CQC-IMS), University of Coimbra, 3004-535 Coimbra, Portugal; mmc.margarida0@gmail.com

[2] Department of Chemistry, University of Coimbra, 3004-535 Coimbra, Portugal

[3] CNC—Centre for Neuroscience Cell Biology, University of Coimbra, UC-Biotech, Parque tecnológico de Cantanhede, núcleo 04, lote 8, 3060-197 Cantanhede, Portugal

[4] Institute for Interdisciplinary Research, University of Coimbra, Casa Costa Alemão, 3030-789, Coimbra, Portugal

* Correspondence: salvador@cnc.uc.pt (A.S.); mmoreno@ci.uc.pt (M.J.M.)

S1 – Abbreviations 2

S2 – Supplementary figures for non-ionisable solutes and weak acids 7

S3 – Model for the permeation of weak bases 8

S1 – Abbreviations

A_{io}	Total area of the surfaces separating the two membrane leaflets
a_{L}	Surface area *per* lipid molecule
BD_{wo}	Deprotonated form of the buffer in the outer aqueous compartment
BH_{wo}	Protonated form of the buffer in the outer aqueous compartment
B_{T}	Concentration of the pH buffer outside the vesicles $(nB_{\mathrm{T}}/V_{\mathrm{wo}})$
β	Characteristic constant
c_{L}	Lipid concentration
$\mathrm{f}SH_{\mathrm{lo}}$	Fraction of protonated acid (the neutral permeating species) in the outer membrane leaflet $(SH_{\mathrm{lo}}/(SH_{\mathrm{lo}}+SD_{\mathrm{lo}}))$
$\mathrm{f}SH_{\mathrm{o}}$	Fraction of protonated acid (the neutral permeating species) in the outer compartment $\left((SH_{\mathrm{lo}}+SH_{\mathrm{wo}})/(SH_{\mathrm{lo}}+SD_{\mathrm{lo}}+SH_{\mathrm{wo}}+SD_{\mathrm{wo}})\right)$
$\mathrm{f}SH_{\mathrm{wD}}$	Fraction of protonated acid (the neutral permeating species) in the donor aqueous compartment $(SH_{\mathrm{wD}}/(SH_{\mathrm{wD}}+SD_{\mathrm{wD}}))$
$\mathrm{f}V_{\mathrm{lA}}$	Fractional volume of the membrane leaflet of the acceptor compartments $(V_{\mathrm{lA}}/V_{\mathrm{T}})$
$\mathrm{f}V_{\mathrm{lD}}$	Fractional volume of the membrane leaflet of the donor compartments $(V_{\mathrm{lD}}/V_{\mathrm{T}})$
$\mathrm{f}V_{\mathrm{li}}$	Fractional volume of the inner membrane leaflet $(V_{\mathrm{li}}/V_{\mathrm{T}})$
$\mathrm{f}V_{\mathrm{lo}}$	Fractional volume of the outer membrane leaflet $(V_{\mathrm{lo}}/V_{\mathrm{T}})$
$\mathrm{f}V_{\mathrm{wA}}$	Fractional volume of the acceptor aqueous compartments $(V_{\mathrm{wA}}/V_{\mathrm{T}})$
$\mathrm{f}V_{\mathrm{wD}}$	Fractional volume of the donor aqueous compartments $(V_{\mathrm{wD}}/V_{\mathrm{T}})$
$\mathrm{f}V_{\mathrm{wi}}$	Fractional volume of the inner aqueous media $(V_{\mathrm{wi}}/V_{\mathrm{T}})$
$\mathrm{f}V_{\mathrm{wo}}$	Fractional volume of the outer aqueous medium $(V_{\mathrm{wo}}/V_{\mathrm{T}})$
h	Membrane thickness
$\boldsymbol{H}_{\mathrm{i}}$	Aggregated variable including free protons and species carrying a labile proton in the inner compartments $(nH_{\mathrm{wi}}+nSH_{\mathrm{wi}}+nSH_{\mathrm{li}}+nPH_{\mathrm{wi}})/V_{\mathrm{i}}$

$\boldsymbol{H}_{\mathbf{o}}$	Aggregated variable including free protons and species carrying a labile proton in the outer compartments $(nH_{\mathrm{wo}}+nSH_{\mathrm{wo}}+nSH_{\mathrm{lo}}+nBH_{\mathrm{wo}})/(V_{\mathrm{wo}}+V_{\mathrm{lo}})$
H_{T}	Total concentration of protons $\left((n\boldsymbol{H}_{\mathbf{o}}+n\boldsymbol{H}_{\mathbf{i}})/V_{\mathrm{T}}\right)$
H_{wi}	Free protons in the inner aqueous compartment
H_{wo}	Free protons in the outer aqueous compartment
$K_{\mathrm{a}}^{\mathrm{B}}$	Acidity constant of the buffer
$K_{\mathrm{a}}^{\mathrm{P}}$	Acidity constant of the fluorescent probe
$K_{\mathrm{a}}^{\mathrm{Sl}}$	Acidity constant of the weak acid in the membrane
$K_{\mathrm{a}}^{\mathrm{Sw}}$	Acidity constant of the weak acid in the aqueous media
$k_{\mathrm{lio}}^{\mathrm{S}}$	Translocation rate constant of the non-ionisable solute towards the outer leaflet
$k_{\mathrm{lio}}^{\mathrm{SH}}$	Translocation rate constant of the weak acid towards the outer leaflet
$k_{\mathrm{loi}}^{\mathrm{S}}$	Translocation rate constant of the non-ionisable solute towards the inner leaflet
$k_{\mathrm{loi}}^{\mathrm{SH}}$	Translocation rate constant of the weak acid towards the inner leaflet
K_{P}	Partition coefficient of the non-ionisable solute between the aqueous media and the membrane
$K_{\mathrm{P}}^{\mathrm{SD}}$	Partition coefficient of the conjugated base between the aqueous media and the membrane
$K_{\mathrm{P}}^{\mathrm{SH}}$	Partition coefficient of the weak acid between the aqueous media and the membrane
$N_{\mathrm{L}\ \mathit{per}\ \mathrm{vesicle}}$	Number of lipids *per* vesicle
N_{vesicles}	Total number of vesicles
P_{app}	$\beta\frac{nS_{\mathrm{A}}(\infty)}{nS_{\mathrm{D}}(0)}\frac{V_{\mathrm{D}}}{A}$ (equation (4) of the main manuscript)
P_{app}^{r}	$\beta\frac{r}{3}$ (equation (6) of the main manuscript)

$P_{\mathrm{app}}^{\mathrm{rV}}$	$\beta \frac{r}{3}\left(1-\frac{V_{\mathrm{A}}}{V_{\mathrm{T}}}\right)$ (equation (19) of the main manuscript)
$P_{\mathrm{app}}^{\mathrm{w}}$	$\beta \frac{nS_{\mathrm{wA}}(\infty)}{nS_{\mathrm{wD}}(0)} \frac{V_{\mathrm{wD}}}{A}$ (equation (21) of the main manuscript)
$P_{\mathrm{app}}^{\mathrm{w*}}$	$\beta \frac{nS_{\mathrm{A}}(\infty)}{nS_{\mathrm{wD}}(0)} \frac{V_{\mathrm{wD}}}{A}$ (equation (24) of the main manuscript)
PD_{wi}	Base form of the fluorescent probe in the inner aqueous compartment
PH_{wi}	Weak acid form of the fluorescent probe in the inner aqueous compartment
P_{obs}	$k_{\mathrm{loi}}^{\mathrm{S}} K_{\mathrm{P}}\ h \frac{1}{\mathrm{f}V_{\mathrm{wD}} + K_{\mathrm{P}}\ \mathrm{f}V_{\mathrm{lD}}}$ (equation (27) of the main manuscript)
P_{T}	Concentration of the fluorescent probe inside the vesicles $(nP_{\mathrm{T}}/V_{\mathrm{wi}})$
r_{i}	Internal vesicle radius $(r-h)$
r_{io}	Radius of the surface separating the two membrane leaflets $(r-h/2)$
r_{o}	Outer vesicle radius
S_{A}	Solute in the acceptor compartment
S_{D}	Solute in the donor compartment
SD_{li}	Solute base form in the inner membrane leaflet
SD_{lo}	Solute base form in the outer membrane leaflet
SD_{wi}	Solute base form in the inner aqueous compartment
SD_{wo}	Solute base form in the outer aqueous compartment
SH_{lD}	Solute acid form in the vicinity of the barrier (donor membrane leaflet)
SH_{li}	Solute acid form in the inner membrane leaflet
SH_{lo}	Solute acid form in the outer membrane leaflet

SH_{wi}	Solute acid form in the inner aqueous compartment
SH_{wo}	Solute acid form in the outer aqueous compartment
$\boldsymbol{S}_{\mathbf{i}}$	Aggregated variable of the internal solute species: $(nSH_{\mathrm{wi}} + nSH_{\mathrm{li}} + nSD_{\mathrm{wi}} + nSD_{\mathrm{li}})/V_{\mathrm{i}}$
S_{lD}	Non-ionisable solute in the donor membrane leaflet
S_{li}	Non-ionisable solute in the inner membrane leaflet
S_{lo}	Non-ionisable solute in the outer membrane leaflet
$\boldsymbol{S}_{\mathbf{o}}$	Aggregated variable of the outer solute species: $(nSH_{\mathrm{wo}} + nSH_{\mathrm{lo}} + nSD_{\mathrm{wo}} + nSD_{\mathrm{lo}})/(V_{\mathrm{wo}} + V_{\mathrm{lo}})$
S_{T}	Total concentration of the solute $(nS_{\mathrm{T}}/V_{\mathrm{T}})$
S_{wA}	Solute in the acceptor aqueous compartment
S_{wD}	Solute in the donor aqueous compartment
S_{wi}	Non-ionisable solute in the inner aqueous compartment
S_{wo}	Non-ionisable solute in the outer aqueous compartment
V_{A}	Total volume of the acceptor compartments
V_{D}	Total volume of the donor compartments
V_{i}	Total volume of the inner compartments $(V_{\mathrm{wi}} + V_{\mathrm{li}})$
$V_{\mathrm{L}\ per\ \mathrm{vesicle}}$	Volume occupied by the lipid molecules in a vesicle
$\overline{V_{\mathrm{L}}}$	Molar volume of the lipid
V_{lA}	Volume of the membrane leaflets of the acceptor compartments
V_{lD}	Volume of the membrane leaflets of the donor compartments
V_{li}	Total volume of the inner leaflets of the vesicles

V_{lo}	Total volume of the outer leaflets of the vesicles
V_T	Total volume $(V_{wo} + V_{lo} + V_{li} + V_{wi})$
V_{wA}	Volume of the acceptor aqueous compartments
V_{wD}	Volume of the donor aqueous compartments
V_{wi}	Total volume of the aqueous medium encapsulated by the vesicles
V_{wo}	Volume of the aqueous compartment outside the vesicles

S2 – Supplementary figures for non-ionisable solutes and weak acids

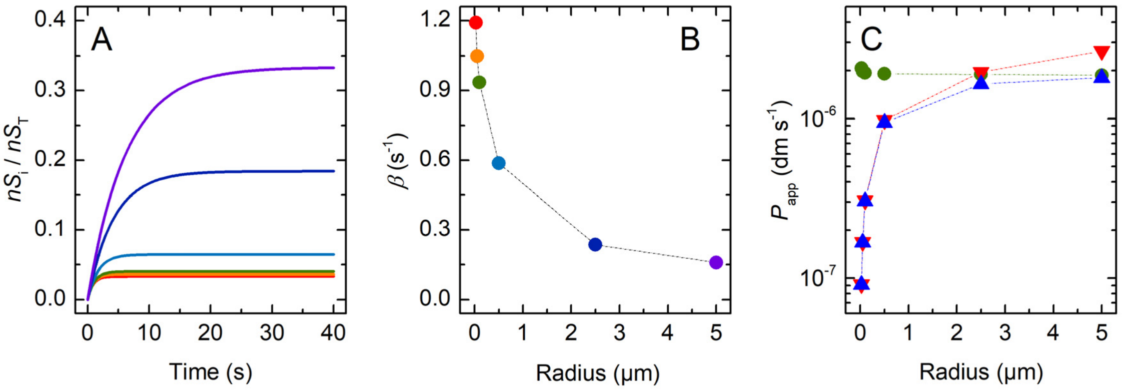

Figure S1 – Permeation of moderately lipophilic non-ionisable solutes, $K_P = 10^2$. (**A**) Variation of the fraction of solute molecules inside the vesicles (membrane inner leaflet *plus* aqueous media) with 25 nm (—), 50 nm (—), 0.1 µm (—), 0.5 µm (—), 2.5 µm (—) and 5 µm radius (—). (**B**) Effect of the vesicle radius on the characteristic constant of solute equilibration. (**C**) Apparent permeability coefficient calculated from the general equation (P_{app} ●), and from the simplified equation (P_{app}^{r} ▼ and P_{app}^{rV} ▲). Note the logarithmic scale of the y axis. The dotted lines in plots B and C are guides for the eye.

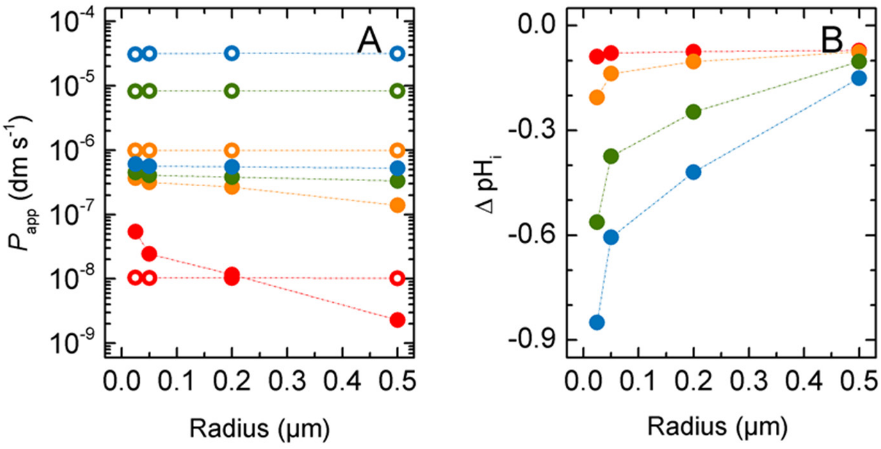

Figure S2 – Dependence of the calculated permeability coefficient (**A**) and observed pH gradient at equilibrium (**B**) on the outer radius of the vesicles, for weak acids with different lipophilicity ($K_P^{SH} = 1$ (●), 10^1 (●), 10^3 (●) and 10^4 (●)). The filled symbols in plot A correspond to P_{app}^{r} and the hollow symbols to P_{app} . The dotted lines are guides for the eye.

S3 – Model for the permeation of weak bases

Figure S3 shows the reaction scheme for the permeation of weak bases. It is considered that

i) the solute acid species are positively charged, and the solute base species have no charges;

ii) only the uncharged (*i.e.*, deprotonated, SD) species permeate. $k_{\text{loi}}^{\text{SD}}$ and $k_{\text{lio}}^{\text{SD}}$ are the translocation rate constants of the weak base towards the inner and outer leaflets, respectively;

iii) protonation and deprotonation steps and partition processes are at quasi-equilibrium. The solute ionisation equilibria are now defined in terms of the acidity constant of the conjugated acid;

iv) the translocation of the weak base is the single rate-limiting step.

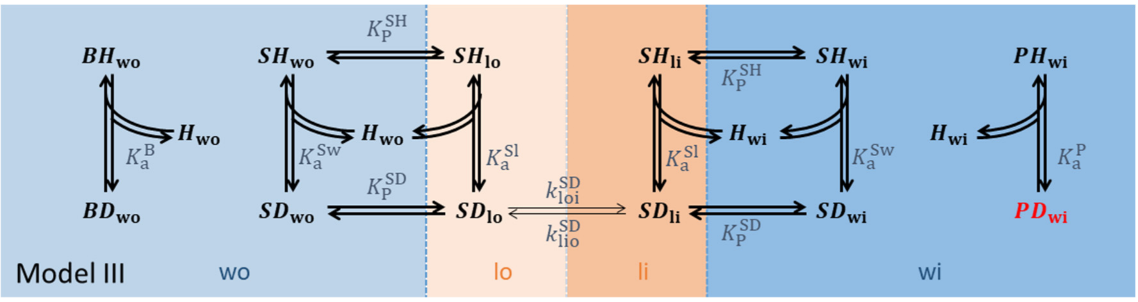

Figure S3 – Reaction scheme for the permeation of a weak base (Model III). The translocation between the membrane leaflets is the single rate-limiting step (thin arrows), with protonation/deprotonation and aqueous/membrane partition processes being at quasi-equilibrium (thick arrows).

For the permeation of a weak base, the differential equation of the aggregated variable $\boldsymbol{S}_{\mathbf{i}}$ is related to the local concentrations of SD_{lo} and SD_{li} as

$$\frac{d\boldsymbol{S}_{\mathbf{i}}(t)}{dt} = -k_{\text{lio}}^{\text{SD}}\, SD_{\text{li}}(t)\frac{V_{\text{li}}}{V_{\text{i}}} + k_{\text{loi}}^{\text{SD}}\, SD_{\text{lo}}(t)\frac{V_{\text{lo}}}{V_{\text{i}}}. \tag{S1}$$

As for the permeation of weak acids, the aggregated variable $\boldsymbol{S}_{\mathbf{i}}$ is related to the local concentrations of the solute as

$$\boldsymbol{S}_{\mathbf{i}}(t) = \left[SH_{\text{wi}}(t) + SD_{\text{wi}}(t)\right]\frac{V_{\text{wi}}}{V_{\text{i}}} + \left[SH_{\text{li}}(t) + SD_{\text{li}}(t)\right]\frac{V_{\text{li}}}{V_{\text{i}}}. \tag{S2}$$

The quasi-equilibrium approximation for the partition into and from the membrane yields the relationships

$$K_{\text{P}}^{\text{SH}} = \frac{SH_{\text{lo}}(t)}{SH_{\text{wo}}(t)} = \frac{SH_{\text{li}}(t)}{SH_{\text{wi}}(t)}; \quad K_{\text{P}}^{\text{SD}} = \frac{SD_{\text{lo}}(t)}{SD_{\text{wo}}(t)} = \frac{SD_{\text{li}}(t)}{SD_{\text{wi}}(t)}, \tag{S3}$$

and for the ionisation of solute, base and probe translates into the relationships

$$K_{\mathrm{a}}^{\mathrm{Sw}} = \frac{SD_{\mathrm{wo}}(t)\, H_{\mathrm{wo}}(t)}{SH_{\mathrm{wo}}(t)} = \frac{SD_{\mathrm{wi}}(t)\, H_{\mathrm{wi}}(t)}{SH_{\mathrm{wi}}(t)}\;;$$
$$K_{\mathrm{a}}^{\mathrm{Sl}} = \frac{SD_{\mathrm{lo}}(t)\, H_{\mathrm{wo}}(t)}{SH_{\mathrm{lo}}(t)} = \frac{SD_{\mathrm{li}}(t)\, H_{\mathrm{wi}}(t)}{SH_{\mathrm{li}}(t)}\;; \tag{S4}$$
$$K_{\mathrm{a}}^{\mathrm{B}} = \frac{BD_{\mathrm{wo}}(t)\, H_{\mathrm{wo}}(t)}{BH_{\mathrm{wo}}(t)}\;;\quad K_{\mathrm{a}}^{\mathrm{P}} = \frac{PD_{\mathrm{wi}}(t)\, H_{\mathrm{wi}}(t)}{BH_{\mathrm{wi}}(t)}.$$

The mass conservation relationships of solute, protons, buffer, and probe are given by:

$$\left[SH_{\mathrm{wo}}(t) + SD_{\mathrm{wo}}(t)\right]\mathrm{f}V_{\mathrm{wo}} + \left[SH_{\mathrm{lo}}(t) + SD_{\mathrm{lo}}(t)\right]\mathrm{f}V_{\mathrm{lo}} + \boldsymbol{S}_{\mathbf{i}}(t)\,\mathrm{f}V_{\mathrm{i}} = S_{\mathrm{T}}$$
$$\left[SH_{\mathrm{wo}}(t) + H_{\mathrm{wo}}(t) + BH_{\mathrm{wo}}(t)\right]\mathrm{f}V_{\mathrm{wo}} + SH_{\mathrm{lo}}(t)\,\mathrm{f}V_{\mathrm{lo}} + \left[SH_{\mathrm{wi}}(t) + H_{\mathrm{wi}}(t) + PH_{\mathrm{wi}}(t)\right]\mathrm{f}V_{\mathrm{wi}} + $$
$$+ SH_{\mathrm{li}}(t)\,\mathrm{f}V_{\mathrm{li}} = H_{\mathrm{T}} \tag{S5}$$
$$BH_{\mathrm{wo}}(t) + BD_{\mathrm{wo}}(t) = B_{\mathrm{T}}$$
$$PH_{\mathrm{wi}}(t) + PD_{\mathrm{wi}}(t) = P_{\mathrm{T}}.$$

Solving the system of equations that includes equations (S3), (S4) and (S5) for the concentration of the species in the outer compartments, as function of the variables $\boldsymbol{S}_{\mathbf{i}}$ and H_{wo} yields the analytical solution:

$$SH_{\mathrm{wo}}(t) = \frac{H_{\mathrm{wo}}(t)\left[S_{\mathrm{T}}\, V_{\mathrm{T}} - \boldsymbol{S}_{\mathbf{i}}(t)\, V_{\mathrm{i}}\right]}{K_{\mathrm{a}}^{\mathrm{Sw}}\left(K_{\mathrm{wlo}}^{\mathrm{SD}}\, V_{\mathrm{lo}} + V_{\mathrm{wo}}\right) + H_{\mathrm{wo}}(t)\left(K_{\mathrm{wlo}}^{\mathrm{SH}}\, V_{\mathrm{lo}} + V_{\mathrm{wo}}\right)}$$
$$SD_{\mathrm{wo}}(t) = \frac{K_{\mathrm{a}}^{\mathrm{Sw}}\left[S_{\mathrm{T}}\, V_{\mathrm{T}} - \boldsymbol{S}_{\mathbf{i}}(t)\, V_{\mathrm{i}}\right]}{K_{\mathrm{a}}^{\mathrm{Sw}}\left(K_{\mathrm{P}}^{\mathrm{SD}}\, V_{\mathrm{lo}} + V_{\mathrm{wo}}\right) + H_{\mathrm{wo}}(t)\left(K_{\mathrm{P}}^{\mathrm{SH}}\, V_{\mathrm{lo}} + V_{\mathrm{wo}}\right)}$$
$$SH_{\mathrm{lo}}(t) = \frac{H_{\mathrm{wo}}(t)\, K_{\mathrm{P}}^{\mathrm{SH}}\left[S_{\mathrm{T}}\, V_{\mathrm{T}} - \boldsymbol{S}_{\mathbf{i}}(t)\, V_{\mathrm{i}}\right]}{K_{\mathrm{a}}^{\mathrm{Sw}}\left(K_{\mathrm{P}}^{\mathrm{SD}}\, V_{\mathrm{lo}} + V_{\mathrm{wo}}\right) + H_{\mathrm{wo}}(t)\left(K_{\mathrm{P}}^{\mathrm{SH}}\, V_{\mathrm{lo}} + V_{\mathrm{wo}}\right)} \tag{S6}$$
$$SD_{\mathrm{lo}}(t) = \frac{K_{\mathrm{a}}^{\mathrm{Sw}}\, K_{\mathrm{P}}^{\mathrm{SH}}\left[S_{\mathrm{T}}\, V_{\mathrm{T}} - \boldsymbol{S}_{\mathbf{i}}(t)\, V_{\mathrm{i}}\right]}{K_{\mathrm{a}}^{\mathrm{Sw}}\left(K_{\mathrm{P}}^{\mathrm{SD}}\, V_{\mathrm{lo}} + V_{\mathrm{wo}}\right) + H_{\mathrm{wo}}(t)\left(K_{\mathrm{P}}^{\mathrm{SH}}\, V_{\mathrm{lo}} + V_{\mathrm{wo}}\right)}$$
$$BH_{\mathrm{wo}}(t) = \frac{B_{\mathrm{T}}\, H_{\mathrm{wo}}(t)}{K_{\mathrm{a}}^{\mathrm{B}} + H_{\mathrm{wo}}(t)}\;;\quad BD_{\mathrm{wo}}(t) = \frac{B_{\mathrm{T}}\, K_{\mathrm{a}}^{\mathrm{B}}}{K_{\mathrm{a}}^{\mathrm{B}} + H_{\mathrm{wo}}(t)}.$$

The overall system of equations lacks a simple closed-form analytical solution and must be solved numerically. This is done considering the partition and ionisation equilibria relationships for the inner solute species and probe, the mass conservations of the solute and the probe. All the required equations for the numerical integration procedure are listed above.
